# Shifting bisphosphonate prescribing patterns for fracture prevention: a 24-year national surveillance of men in the U.S. Veterans Health Administration

**DOI:** 10.1007/s11657-025-01635-z

**Published:** 2026-01-08

**Authors:** Rachel E. Elam, Joan C. Lo, John T. Schousboe, Robert A. Adler, Howard A. Fink, Susan M. Ott, Joshua Barzilay, Frances M. Weaver, Emily Budde, Zhiping Huo, Reside Jacob, Laura D. Carbone

**Affiliations:** 1 Division of Rheumatology, Department of Medicine, Medical College of Georgia at Augusta University, Augusta, GA USA; 2 Center of Innovation for Complex Chronic Healthcare (CINCCH) Hines VA Hospital, Hines, IL USA; 3 Division of Research, Kaiser Permanente Northern California, 4480 Hacienda Drive, Pleasanton, CA USA; 4 The Permanente Medical Group, Oakland, CA USA; 5 Department of Health Systems Science, Kaiser Permanente Bernard J. Tyson School of Medicine, Pasadena, CA USA; 6 HealthPartners Institute, HealthPartners, Inc., Bloomington, MN USA; 7 Division of Health Policy and Management, University of Minnesota, Minneapolis, MN USA; 8 Richmond Veterans Affairs Medical Center and Virginia Commonwealth University, Richmond, VA USA; 9 Geriatric Research Education and Clinical Center, Veterans Affairs Healthcare System, Minneapolis, MN USA; 10 Department of Medicine, University of Washington School of Medicine, Seattle, WA USA; 11 Center for Research and Evaluation, Kaiser Permanente Georgia and Division of Endocrinology, Southeast Permanente Medical Group, Atlanta, GA USA; 12 Professor Emeritus, Parkinson School of Health Sciences and Public Health, Loyola University, Maywood, IL USA; 13 Harold Harrison, MD. Distinguished University Chair in Rheumatology, Division of Rheumatology, Department of Medicine, Medical College of Georgia at Augusta University, Augusta, GA USA

**Keywords:** Epidemiology, Osteoporosis, Bisphosphonates, Fracture, Age, Frailty

## Abstract

***Summary*:**

In the U.S’s largest integrated health system, during a 24-year period (1999–2022), bisphosphonate treatment initiation for fracture prevention in men shifted towards higher-risk populations, including older men and those with prior fracture and frailty.

**Purpose:**

To evaluate 24-year trends in bisphosphonate (BP) initiation among older U.S. male Veterans and shifts in demographic and clinical characteristics of BP-treated men over time.

**Methods:**

U.S. national Veterans Health Administration (VHA) data (1999–2022) were queried to identify men aged ≥ 50 years with a first prescription for an FDA-approved BP for fracture prevention. Age, race, ethnicity, BP drug and route, prior fracture, and, in those aged ≥ 65 years, Veterans Affairs Frailty Index (VA-FI), were examined across five time periods. Temporal trends were analyzed using chi-square and nonparametric trend tests.

**Results:**

A total of 298,340 men initiated a BP during 1999–2022, of whom 233,857 (78.4%) were aged ≥ 65 years. BP initiation rose sharply after FDA approval of BPs for men in 2000, peaked in 2004–2005, then declined by about 50% between 2006 and 2012, and then plateaued. Over time, the proportion of BP initiators aged < 65 years declined from a peak of 28.2% during the middle time period (2008–2012) to a nadir of 13.3% during the final years (2018–2022, *p* < 0.001 for trend). Among the subset of men age 65 and older who initiated BP, the proportion with prior fracture increased from 8.3% in 1999–2002 to 24.5% in 2018–2022 (*p* < 0.001). Notably, over half of the men who initiated BP during 1999–2002 were classified as non-frail, whereas in the most recent time period (2018–2022), over half of BP initiators were frail (mildly, moderately, or severely) and only 14.8% of them were non-frail (*p* < 0.001).

**Conclusion:**

In the VHA, BP initiating patterns shifted over time towards treating older men, with much larger proportions of men who had a prior fracture and were classified as frail.

**Supplementary Information:**

The online version contains supplementary material available at 10.1007/s11657-025-01635-z.

## Introduction

Multiple randomized clinical trials, predominately in postmenopausal women, demonstrate that oral or intravenous (IV) bisphosphonate (BP) therapy following a hip or vertebral fracture substantially reduces subsequent fractures[1, 2]. Although there is less evidence for fracture risk reduction in older men with osteoporosis than for older women, it is generally thought that these drugs have similar efficacy as in women[3] and that men with a high risk of fracture should be treated[4].

In 2000, alendronate became the first BP approved for the treatment of osteoporosis in men. BPs were available since 1995 for the treatment of postmenopausal osteoporosis in women, and prior to 2002, treatment of women younger than age 65 with BPs for fracture prevention was common[5]. Following this, a number of guidelines were published suggesting that older women be treated for osteoporosis, although some made no specific recommendations for men[6, 7], while others[8–10] recommended treatment for men age 50 and older with *T*-scores < −2.5 or those with low bone mass or osteopenia (*T*-score −1 to −2.5) who had a high 10-year probability of hip or major osteoporotic fracture, as determined by FRAX[8]—a fracture risk assessment tool introduced in 2008[9].

In the time period between 2003 and 2005, warnings about the risks of bisphosphonate-associated osteonecrosis of the jaw (BONJ) were disseminated by national regulatory agencies, the manufacturers of bisphosphonates, and the International Myeloma Foundation[11], based on early reports in cancer patients receiving high-frequency IV BP therapy. In 2005, the U.S. Food and Drug Administration (FDA) began requiring that all BP drug labels include warnings about the risk of BONJ[11]. Shortly after, case series of unusual fractures occurring in the subtrochanteric and diaphyseal femur region arising after long-term BP treatment for osteoporosis emerged in 2005–2007[12]. In October 2010, the FDA issued a drug safety communication regarding risks of atypical femoral fractures (AFFs) following BP therapy for osteoporosis. This information was subsequently added to the warnings and precautions sections of the labels of all BP drugs approved for the prevention or treatment of osteoporosis. Following recognition of these late adverse effects from BP therapies, BP use in the U.S.A. declined by more than 50% between 2008 and 2012, despite earlier increases for more than a decade[13, 14].

However, premature discontinuation of BPs, particularly in older individuals at higher risk for fracture, is associated with increased fracture risk[15]. Some[16, 17], but not all[18], studies suggest that targeted strategies such as fracture liaison services increase the number of individuals treated with BP therapy for osteoporosis. Lo et al. reported that implementing a BMD screening metric in the electronic health record (EHR) of a single, large U.S. integrated health system was temporally associated with a sustained number of women initiating BP therapy and a large increase among men, a population previously not targeted by regional primary fracture prevention initiatives[19]. Dell et al. also highlighted the important role of osteoporosis screening and observed parallel trends in screening and treatment[20]. Within the Veterans Health Administration (VHA), the largest integrated healthcare system in the U.S.A., programs aimed at increasing awareness of and treatment for osteoporosis, predominantly in men, have existed since 2012, but many of these were only done at local, and not national, levels [21, 22].

## Purpose

The purpose of this report was to examine temporal trends in BP initiation among men in the VHA from 1999 to 2022, to determine the temporal association of reporting adverse events from BPs and osteoporosis initiatives on BP initiation rates and to describe the characteristics of men who were treated over different time periods.

## Methods

### Study population

Participants were identified from the Veterans Health Administration (VHA) Corporate Data Warehouse (CDW). They included male veterans aged 50 years and older with at least one filled VA prescription for an FDA-approved oral or IV BP for fracture prevention from October 1, 1999, through December 31, 2022. Those veterans with a history of secondary metastatic cancer (International Classification of Diseases (ICD)−9 codes 197X, 198.X, and 199.0; ICD-10 codes C78.X, C79.X, and C80.X), multiple myeloma (ICD-9 code 203.0X; ICD-10 code C90.0X), Paget’s disease of bone (ICD-9 code 731.0; ICD-10 code M88.X), osteogenesis imperfecta (ICD-9 code 756.51; ICD-10 code Q78.0), hypophosphatasia or phosphate wasting syndromes (ICD-9 code 275.3; ICD-10 code E83.3X), osteopetrosis (ICD-9 code 756.52; ICD-10 code Q78.2), and end-stage renal disease (ESRD) or renal failure (ICD-9 code 585.6; ICD-10 N18.6) were excluded. Those with a filled prescription at any time during the study period for etidronate, pamidronate, or tiludronate and those whose dose of zoledronic acid was or ≤ 273 days from the initial dose were also excluded, as their BP was unlikely to have been prescribed for fracture prevention.

The study was declared exempt by the VA central Institutional Review Board (IRB) and was performed in accordance with the ethical standards as laid down in the 1964 Declaration of Helsinki and its later amendments.

### Participant characteristics

Demographic characteristics were ascertained at the date of or closest to the date prior to the first filled BP prescription and included age, race, and ethnicity. Prior clinical fracture (excluding head/face, fingers, and toes) during the 5 years preceding BP initiation was ascertained (ICD-9 codes 733.1, 805.X, 807–829.X; ICD-10 codes M48.4, M48.5, M80, M84.4, M84.6, S12.X, S22.X, S32.X, S43.X, S52.X, S62.X,S72.X, S82.X, S92.X). Medication details were also captured, including the agent and route of BP initiated. The Veterans Affairs Frailty Index (VA-FI), which uses up to 31 health deficits to assess frailty on a score of 0 to 1, was categorized into five groups as follows: non-frail, pre-frail, mildly frail, moderately frail, severely frail, and was ascertained at the date of or closest to the date of the first filled BP prescription among those men age 65 and older[23].

### Statistical analyses

Descriptive statistics, including means/standard deviations and proportions, were determined across five historically meaningful time periods (1999–2002, 2003–2007, 2008–2012, 2013–2017, 2018–2022). Dividing the 24-year period into these 4-year segments was done to examine potentially meaningful time periods as a group. For example, 2003–2007 was the time period in which adverse events from BPs were first reported. The time period of 2008–2012 was the period in which across the U.S. BP prescriptions fell after reports of these adverse events. Differences by time periods in treatment patterns and the association of specific BP agent, BP route, age (and age group), race, ethnicity, prior clinical fracture, and VA-FI by time periods (1999–2022) were examined using chi-square tests or tests for trends, as appropriate. The Cochrane-Armitage test for trend was used for binary covariates, and the Jonckheere-Terpstra test for trend was used for continuous and ordinal variables. In a pre-specified subgroup analysis, the above descriptive statistics and statistical testing for associations and/or trends were performed in men restricted to age 65 and older. We tested for collinearity of prior fractures with frailty categories from the VA-FI in men age 65 and older by calculating a mean variance inflation factor in regression models between these two variables. Analyses were performed in STATA version 18.0. A *p*-value of < 0.05 was considered statistically significant.

## Results

**Fig. 1:**
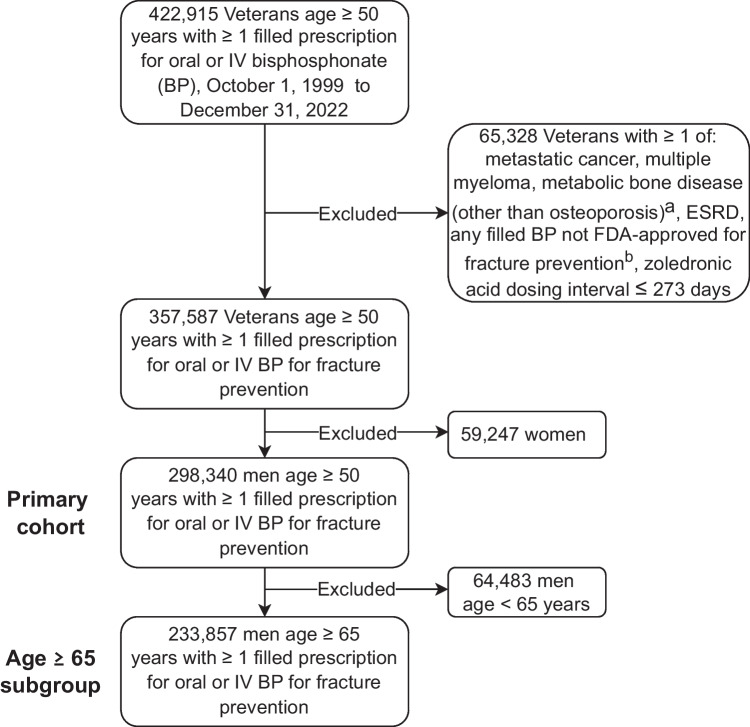
Study flow diagram. IV, intravenous; BP, bisphosphonate; ESRD, end-stage renal disease; FDA, Food and Drug Administration. ^a^Metabolic bone diseases (other than osteoporosis) include Paget’s disease of bone, osteogenesis imperfecta, hypophosphatasia or phosphate wasting syndromes, or osteopetrosis. ^b^BPs not FDA-approved for fracture prevention include the following: pamidronate, tiludronate, and etidronate

There were 298,340 men age 50 years and older who newly initiated BP therapy over the study period and met inclusion criteria (Fig. 1). Among all men who initiated BPs over the 24-year period, 15.3%, 36.3%, 22.4%, 14.3%, and 11.9% were from each respective time period (1999–2002, 2003–2007, 2008–2012, 2013–2017, and 2018–2022). Most initiated oral BP (96.5%), specifically alendronate. The percentage initiating treatment with an IV BP was highest during the most recent time period (2018–2022) at 13.8%.

Table 1 depicts the demographic and clinical characteristics of the study population at BP initiation across successive time periods. The proportion aged < 65 years peaked in the middle time period (2008–2012) at 28.2% and then subsequently declined to a nadir in 2018–2022 at 13.3% (*p* < 0.001 for trend). The racial composition of men initiating BP varied over time (*p* < 0.001), with higher proportions of American Indian/Alaska Native (0.2% vs 0.6%), Asian (0.3% vs 0.7%), Black (3.0% vs 8.9%), Native Hawaiian/Pacific Islander (0.5% vs 0.8%), and multiracial (0.4% vs 0.7%) groups among men initiating BP in the most recent time period (2018–2022) versus the earliest (1999–2002). A rise in the proportion of Hispanic/Latino men among those who initiated BP over more recent time periods was also observed (*p* < 0.001 for trend).

**Table 1:** Characteristics of adults aged ≥ 50 years initiating bisphosphonate (BP) therapy by time periods

	1999–2002	2003–2007	2008–2012	2013–2017	2018–2022	*P* value^a^
MEN (*N* = 298,340)	*N* = 45,519	*N* = 108,184	*N* = 66,752	*N* = 42,449	*N* = 35,436	—
Age (years), mean ± SD	72.2 ± 8.9	73.3 ± 9.7	72.6 ± 10.5	72.6 ± 9.8	74.0 ± 8.7	< 0.001
Age group (years)						< 0.001
50–64	8,837 (19.4)	23,660 (21.9)	18,793 (28.2)	8,478 (20.0)	4,715 (13.3)	
≥ 65	36,682 (80.6)	84,524 (78.1)	47,959 (71.8)	33,971 (80.0)	30,721 (86.7)	
Race						< 0.001
American Indian/Alaskan Native	86 (0.2)	291 (0.3)	279 (0.4)	263 (0.6)	224 (0.6)	
Asian	114 (0.3)	492 (0.5)	397 (0.6)	243 (0.6)	251 (0.7)	
Black	1,369 (3.0)	5,374 (5.0)	5,479 (8.2)	3,968 (9.4)	3,139 (8.9)	
Native Hawaiian/Pacific Islander	216 (0.5)	700 (0.7)	439 (0.7)	269 (0.6)	271 (0.8)	
White	26,083 (57.3)	81,944 (75.8)	54,396 (81.5)	35,537 (83.7)	29,636 (83.6)	
Multiracial	166 (0.4)	549 (0.5)	388 (0.6)	283 (0.7)	238 (0.7)	
Other/Unknown	17,485 (38.4)	18,834 (17.4)	5,374 (8.1)	1,886 (4.4)	1,677 (4.7)	
Ethnicity						< 0.001
Hispanic/Latinx	817 (1.8)	4,094 (3.8)	3,338 (5.0)	2,526 (6.0)	2,009 (5.7)	
Not Hispanic/Latinx	26,624 (58.5)	86,306 (79.8)	58,979 (88.4)	38,227 (90.1)	31,918 (90.1)	
Unknown	18,078 (39.7)	17,784 (16.4)	4,435 (6.6)	1,696 (4.0)	1,509 (4.3)	
Prior Clinical Fracture	3,982 (8.8)	14,435 (13.3)	12,506 (18.7)	10,665 (25.1)	8,755 (24.7)	< 0.001
First BP route initiated						< 0.001
Oral	45,495 (100)	107,714 (99.6)	65,068 (97.5)	38,923 (91.7)	30,565 (86.3)	
Intravenous	24 (0.1)	470 (0.4)	1,684 (2.5)	3,526 (8.3)	4,871 (13.8)	
First BP agent initiated						< 0.001
Alendronate	44,621 (98.0)	101,078 (93.4)	62,507 (93.6)	38,170 (89.9)	29,993 (84.6)	
Ibandronate^b^	0 (0)	38 (0.0)	54 (0.1)	35 (0.1)	20 (0.1)	
Risedronate	874 (1.9)	6,607 (6.1)	2,518 (3.8)	720 (1.7)	552 (1.6)	
Zoledronic acid	24 (0.1)	461 (0.4)	1,673 (2.5)	3,524 (8.3)	4,871 (13.8)	

*SD* Standard deviation; *BP* bisphosphonate

^a^Cochran-Armitage test for trend for binary variables, Jonckheere-Terpstra test for trend for continuous and ordinal variables, and χ^2^ test for association for categorical, nonordinal variables

^b^Includes both oral and intravenous formulations of ibandronate

**Fig. 2:**
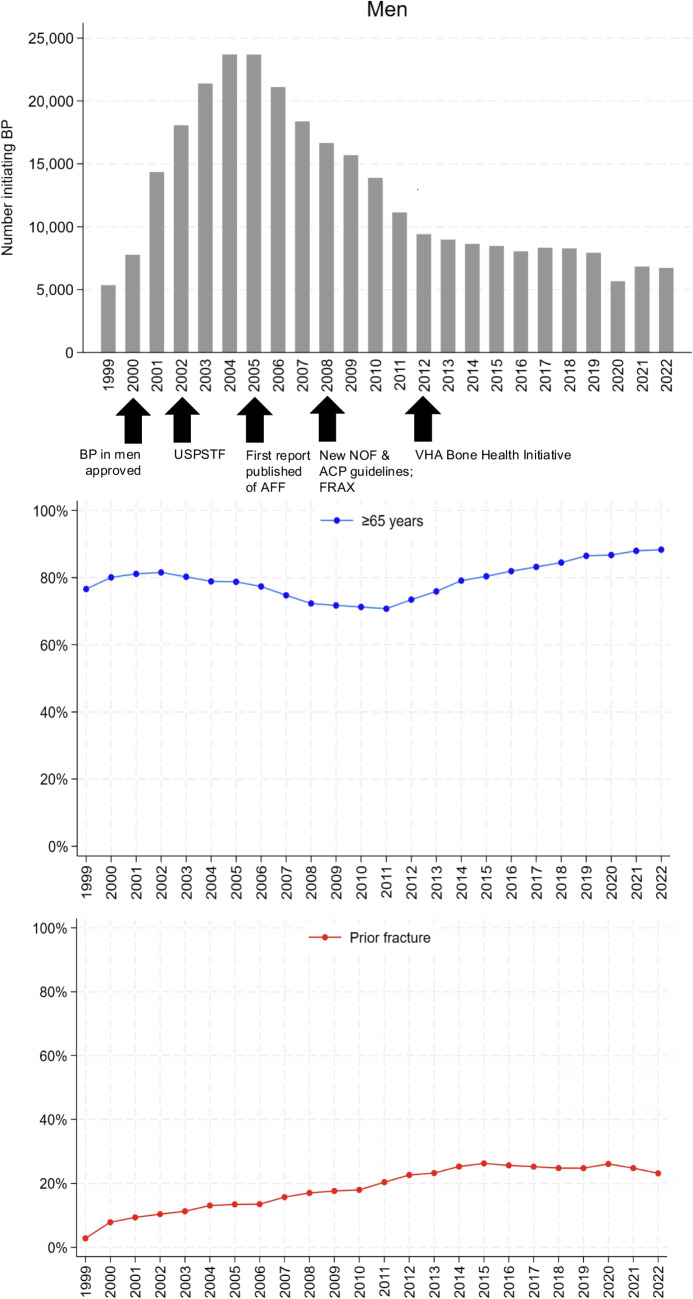
Men age $\ge$ 50 years initiating bisphosphonate (BP) therapy each year and the percentages who were aged $\ge$ 65 years and had prior fracture. USPSTF, United States Preventive Services Task Force; AFF, atypical femoral fracture; NOF, National Osteoporosis Foundation (now the Bone Health & Osteoporosis Foundation since 2021); ACP, American College of Physicians; FDA, Food and drug administration; VHA, Veterans Health Administration. Black arrows mark timing of BP approval in men; USPSTF[6] and new NOF and ACP guidelines[10] and FRAX[9]; first published report of AFF secondary to BP; and onset of the VHA Bone Health Initiative

Figure 2 shows the yearly trends among men initiating BP therapy during 1999–2022, including counts and the proportions by age (≥ 65 years) and prior fracture, with key timepoints in national bone health and osteoporosis care initiatives at the VHA indicated. There was a rapid rise in BP initiation following the FDA approval of BPs in men in 2000, peaking in 2004–2005. BP initiation in men rapidly declined following reports of AFF from 2006 to 2012, with a relative plateauing thereafter. The proportion of men with prior fracture among those who initiated BP steadily rose until around 2015 and then plateaued.

**Fig. 3:**
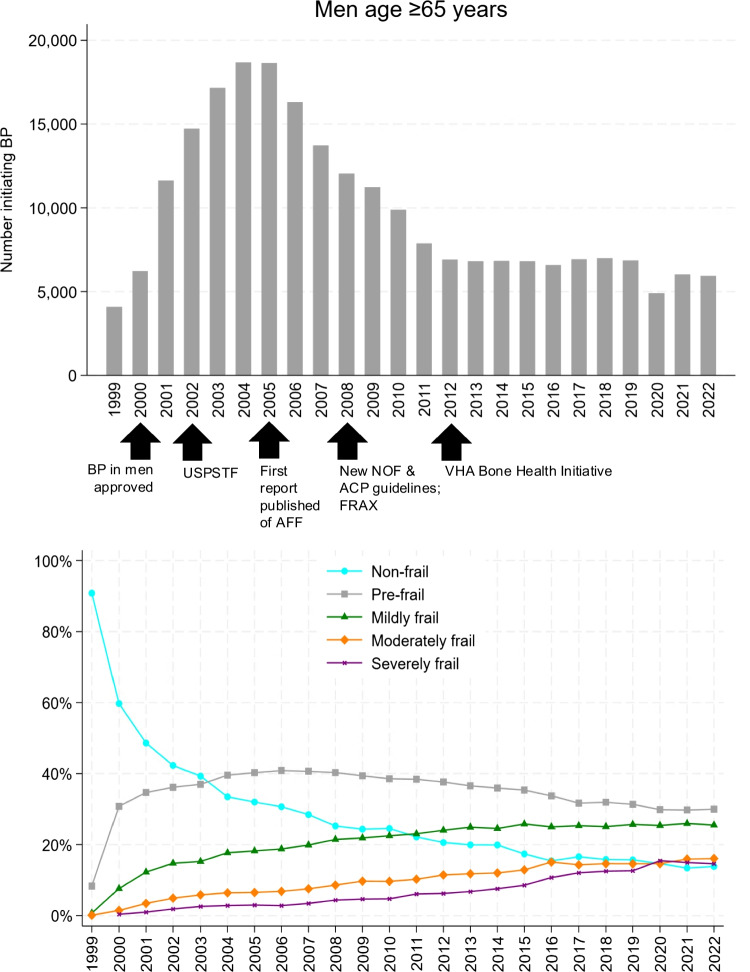
Men age $\ge$ 65 years initiating bisphosphonate (BP) therapy each year and the annual distribution according to the VA-FI. USPSTF, United States Preventive Services Task Force; AFF, atypical femoral fracture; NOF, National Osteoporosis Foundation (now the Bone Health & Osteoporosis Foundation since 2021); ACP, American College of Physicians; FDA, Food and drug administration; VHA, Veterans Health Administration. Black arrows mark timing of BP approval in men; USPSTF[6] and new NOF and ACP guidelines^10^ and FRAX^9^; first published report of AFF secondary to BP; and onset of the VHA Bone Health Initiative

Among men age ≥ 65 years who initiated BPs over the 24-year period, 15.7%, 36.1%, 20.5%, 14.5%, and 13.1% were from each respective time period (1999–2002, 2003–2007, 2008–2012, 2013–2017, and 2018–2022). Among men age ≥ 65 years, BP initiation rose over time in those aged 65–74 years old and ≥ 90 years old (*p* < 0.001 for trend). In the age ≥ 65 subgroup, differences by time periods in treatment patterns and associations of specific BP agents, BP routes, race, ethnicity, and prior clinical fracture were similar to the group as a whole (*p* < 0.001 for all; Table 2). Compared to non-frail individuals, new BP initiation was more common in mildly, moderately, and severely frail individuals over the study period (*p* < 0.001 for trend; Table 2). In the first time period (1999–2002), over half of men initiating BP were non-frail, whereas in the most recent time period (2018–2022), over half were frail (mildly, moderately, or severely) and only 14.8% of men initiating BP were non-frail (Table 2, Fig. 3).

**Table 2:** Characteristics of adults aged ≥65 years initiating bisphosphonate (BP) therapy by time periods

	1999–2002	2003–2007	2008–2012	2013–2017	2018–2022	*P* value^a^
MEN (*N* = 233,857)	36,682	84,524	47,959	33,971	30,721	—
Age (years), mean ± SD	75.7 ± 5.8	77.4 ± 6.4	77.6 ± 7.5	75.8 ± 8.0	76.2 ± 6.9	< 0.001
Age group (years)						< 0.001
65 – 74	15,353 (41.9)	28,803 (34.1)	16,784 (35.0)	17,317 (51.0)	14,723 (47.9)	
75 – 89	21,006 (57.3)	54,078 (64.0)	28,880 (60.2)	14,572 (42.9)	14,508 (47.2)	
≥ 90	323 (0.9)	1,643 (1.9)	2,295 (4.8)	2,082 (6.1)	1,490 (4.9)	
Race						< 0.001
American Indian/Alaskan Native	49 (0.1)	163 (0.2)	157 (0.3)	191 (0.6)	192 (0.6)	
Asian	90 (0.3)	392 (0.5)	326 (0.7)	202 (0.6)	212 (0.7)	
Black	878 (2.4)	3,393 (4.0)	3,259 (6.8)	2,657 (7.8)	2,409 (7.8)	
Native Hawaiian/Pacific Islander	161 (0.4)	518 (0.6)	315 (0.7)	213 (0.6)	325 (0.8)	
White	19,785 (53.9)	62,739 (74.2)	39,173 (81.7)	28,921 (85.1)	26,035 (84.8)	
Multiracial	108 (0.3)	367 (0.4)	266 (0.6)	214 (0.6)	192 (0.6)	
Other/Unknown	15,611 (42.6)	16,952 (20.1)	4,463 (9.3)	1,573 (4.6)	1,446 (4.7)	
Ethnicity						< 0.001
Hispanic/Latinx	591 (1.6)	3,063 (3.6)	2,415 (5.0)	1,900 (5.6)	9,621 (5.4)	
Not Hispanic/Latinx	20,106 (54.8)	65,740 (77.8)	42,007 (87.6)	30,705 (90.4)	27,769 (90.4)	
Unknown	15,985 (43.6)	15.721 (18.6)	3,537 (7.4)	1,366 (4.0)	1,300 (4.2)	
Prior Clinical Fracture	3,040 (8.3)	10,320 (12.2)	8,364 (17.4)	8,330 (24.5	7,527 (24.5)	
First BP route initiated						< 0.001
Oral	36,665 (100)	84,218 (99.6)	46,819 (97.6)	31,219 (91.9)	26,532 (86.4)	
Intravenous	17 (0.1)	306 (0.4)	1,140 (2.4)	2,752 (8.1)	4,189 (13.6)	
First BP agent initiated						< 0.001
Alendronate	35,978 (98.1)	78,824 (93.3)	44,842 (93.5)	30,615 (90.1)	26,029 (84.7)	
Ibandronate^b^	0 (0)	27 (0.0)	42 (0.1)	23 (0.1)	17 (0.1)	
Risedronate	687 (1.9)	5,374 (6.4)	1,944 (4.1)	579 (1.7)	486 (1.6)	
Zoledronic acid	17 (0.1)	299 (0.4)	1,131 (2.4)	2,750 (8.1)	4,189 (13.6)	
Frailty Index Category						< 0.001
Non-frail	19,323 (52.7)	27,868 (33.0)	11,379 (23.7)	6,069 (17.9)	4,533 (14.8)	
Pre-frail	11,620 (31.7)	33,493 (39.6)	18,720 (39.0)	11,772 (34.7)	9,418 (30.7)	
Mildly frail	4,103 (11.2)	15,123 (17.9)	10,750 (22.4)	8,536 (25.1)	7,839 (25.5)	
Moderately frail	1,220 (3.3)	5,570 (6.6)	4,680 (9.8)	4,488 (13.2)	4,659 (15.2)	
Severely frail	416 (1.1)	2,470 (2.9)	2,430 (5.1)	3,106 (9.1)	4,272 (13.9)	

*SD* Standard deviation; *BP* bisphosphonate

^a^Cochran-Armitage test for trend for binary variables, Jonckheere-Terpstra test for trend for continuous and ordinal variables, and χ^2^ test of independence for association for categorical, nonordinal variables

^b^Includes both oral and intravenous formulations of ibandronate

The mean variance inflation factor in regression models between prior fracture history and VA-FI was 1.1, indicating low collinearity.

## Discussion

Significant trends in BP initiation in older men were noted over the 24-year observation period for this study, with the number of men initiating BP peaking in 2004–2005, followed by a rapid decline from 2006 to 2012, and plateauing thereafter. There was a clear shift over time in the characteristics of men who did initiate BPs, with initiation increasing in later years in men at higher risk for fracture, including older men, those with a prior fracture, and/or men who were frail.

In the U.S.A., BP use also declined by more than 50% between 2008 and 2012, despite earlier increases for more than a decade[13, 14]. In male veterans, the steep rise in BP initiation during 2002–2005 occurred after initial FDA approval in 2000 for use in men and began to decline in late 2005–2006, coinciding with the FDA mandate requiring that all bisphosphonate drug labels include warnings about the risk of osteonecrosis of the jaw[11] and published case series of atypical femur fractures arising after long-term BP treatment for osteoporosis[12]. One healthcare system observed relatively sustained numbers of women and men initiating BP treatment during 2008–2012, potentially due to large scale efforts targeting secondary and primary prevention of fracture in women[19]. This same study showed relatively low but stable rates of BP initiation in men, until an electronic health record metric was implemented in 2017, which coincided with larger numbers initiating BP treatment, with some reduction during the COVID-19 pandemic[19]. We likewise saw lower numbers of new BP initiators in 2020 compared to the overall trend, presumably due to the COVID-19 pandemic’s impact, resulting in temporary, large reductions in in-person care in the VHA[24].

National bone health initiatives and, to a lesser extent, VHA initiatives to promote screening and treatment for osteoporosis in men were not temporally associated with increases in BP initiation. In 2008, revised osteoporosis treatment guidelines from the National Osteoporosis Foundation (now the Bone Health and Osteoporosis Foundation), new treatment guidelines from the American College of Physicians (ACP)[10, 25], and widespread adoption of the FRAX[9] tool shifted treatment efforts towards older adults and those with fractures, trends also observed in our cohort. The 2012 VHA Bone Health Initiative represented a strategic shift towards preventive, evidence-based screening and treatment for osteoporosis in male veterans but was not temporally associated with an increase in BP initiation. This may be for several reasons, including that initiatives were limited to local and not national VHAs, failure to disseminate this information, or failure of utilization of the information. In the case of the VHA Bone Health Initiative, in 2012 and again in 2014, there were undersecretary letters drafted by Adler et al. that were distributed to each of the VHA facilities[26]. The aim was to increase treatment of patients, especially men, at high risk for fractures and included those with a new onset vertebral fracture or those who underwent hip fracture surgery at the VHA within the prior 6 months. Other high-risk groups targeted included men on oral glucocorticoids for > 3 months and men on androgen deprivation therapy. However, these letters were not widely disseminated (personal communication Adler). More recently, passive VHA initiatives have become available for all VHA providers involved in osteoporosis management, including access to an Osteoporosis Screening and Management Toolkit (implemented in 2020) and a national clinical reminder system (implemented in 2021); both are accessed through the EHR (Computerized Patient Record System (CPRS)). However, neither of these initiatives was associated with increased BP initiation in our study. This may be secondary to “alert fatigue,” as many physicians may receive more than 50 alerts per day through CPRS at the VHA[27] or the impact of the COVID-19 pandemic on access to care in general[24]. It remains to be seen how the 2024 VHA Policy and Practice Support Age-Specific Osteoporosis Screening in Women initiative will fare, given its focus on women, in contrast to prior initiatives, which mainly targeted men.

The importance of properly directing osteoporosis treatments to populations at highest risk for fracture, including older persons[28], persons with osteoporosis[29], and those with prevalent fracture[30] is now recognized. In the VHA, we observed a rise in BP initiation in men who were 65 years and older in 2011 and/or with prevalent clinical fracture. Likewise, Lo et al. reported a substantial shift after 2008 towards treatment with BPs for both men and women who were 65 and older and/or who had prevalent fractures[19].

We also observed that BP initiation over time was shifted to men who were frail.

To our knowledge, prior reports examining trends in BP initiation have not considered the association of frailty. Following a hip fracture, frail patients have a higher risk of mortality compared to non-frail individuals[31]. Moreover, pharmacological treatments to prevent fracture appear to be as efficacious in frail persons as in those without frailty[32]. Our findings in older men that a higher proportion of BP starts over time occurring in those with mild, moderate, and severe frailty indicate a potential growing recognition of the importance of frailty as a risk factor for fracture and its complications in men.

The racial and ethnic distribution of men initiating BP shifted significantly over time in the VHA during our study period, with greater proportions of BP initiators who were Black and Hispanic in the most recent period compared with the earlier periods. There is evidence to suggest that racial and ethnic disparities have existed in the provision of osteoporosis treatments for fracture prevention[33–36]. In postmenopausal women with osteoporosis, studies have reported that Black women are less likely to be treated for fracture prevention than White women[33–36]. Although there is less evidence in men, significant racial differences in treatment rates among men with hip fractures have been reported, with one study reporting that non-Hispanic Black and Asian men had significantly lower treatment rates (11.1% and 13.3%) than Hispanic (22.5%) and non-Hispanic White men (35.0%)[35]. Future studies should assess whether there are racial and/or ethnic differences in the proportion of men who are at high risk for future fracture based on having a prevalent fracture, in terms of being appropriately targeted for DXA testing and provision of pharmacological therapies to reduce subsequent fractures, particularly given trends in rising fracture rates in these populations[37].

We acknowledge a number of limitations of our work. We excluded non-FDA-approved BPs, such as etidronate; it is possible that these BPs were being used for fracture prevention in the early years of this analysis. Similarly, BP initiation may be blunted in later years with the use of other osteoporosis drugs such as denosumab, teriparatide, abaloparatide, and romosozumab. It is also difficult to know whether the trends in BP initiation we noted over time are related to true differences in BP prescribing patterns or simply reflect differences in the VHA population as a whole, which is becoming older and more racially and ethnically diverse over time. Finally, we cannot be certain that any of the reports of adverse events from BPs or bone initiatives were the cause of changes in BP initiation.

## Conclusion

In conclusion, we noted several substantial temporal trends in BP initiation in men in the largest integrated U.S. healthcare system from 1999 to 2022, particularly by age, prior clinical fracture, and frailty. The populations initiating BP therapy in the VHA had increasing proportions of men who were older, had a prior fracture, or were frail. Future studies should investigate how VHA-specific and more general bone health initiatives influence adherence to and persistence with BP treatment.

## Supplementary Information

Below is the link to the electronic supplementary material.

ESM 1 (PDF 286 KB)

ESM 2 (PDF 226 KB)

## Data Availability

An anonymized dataset is available upon reasonable request after an appropriate DUA.
