# Supplementary figures and images for: Shifting bisphosphonate prescribing patterns for fracture prevention: a 24-year national surveillance of men in the U.S. Veterans Health Administration
Source: Arch Osteoporos. 2026 Jan 8;21(1):20. doi: 10.1007/s11657-025-01635-z (PMC12783237; doi:10.1007/s11657-025-01635-z)

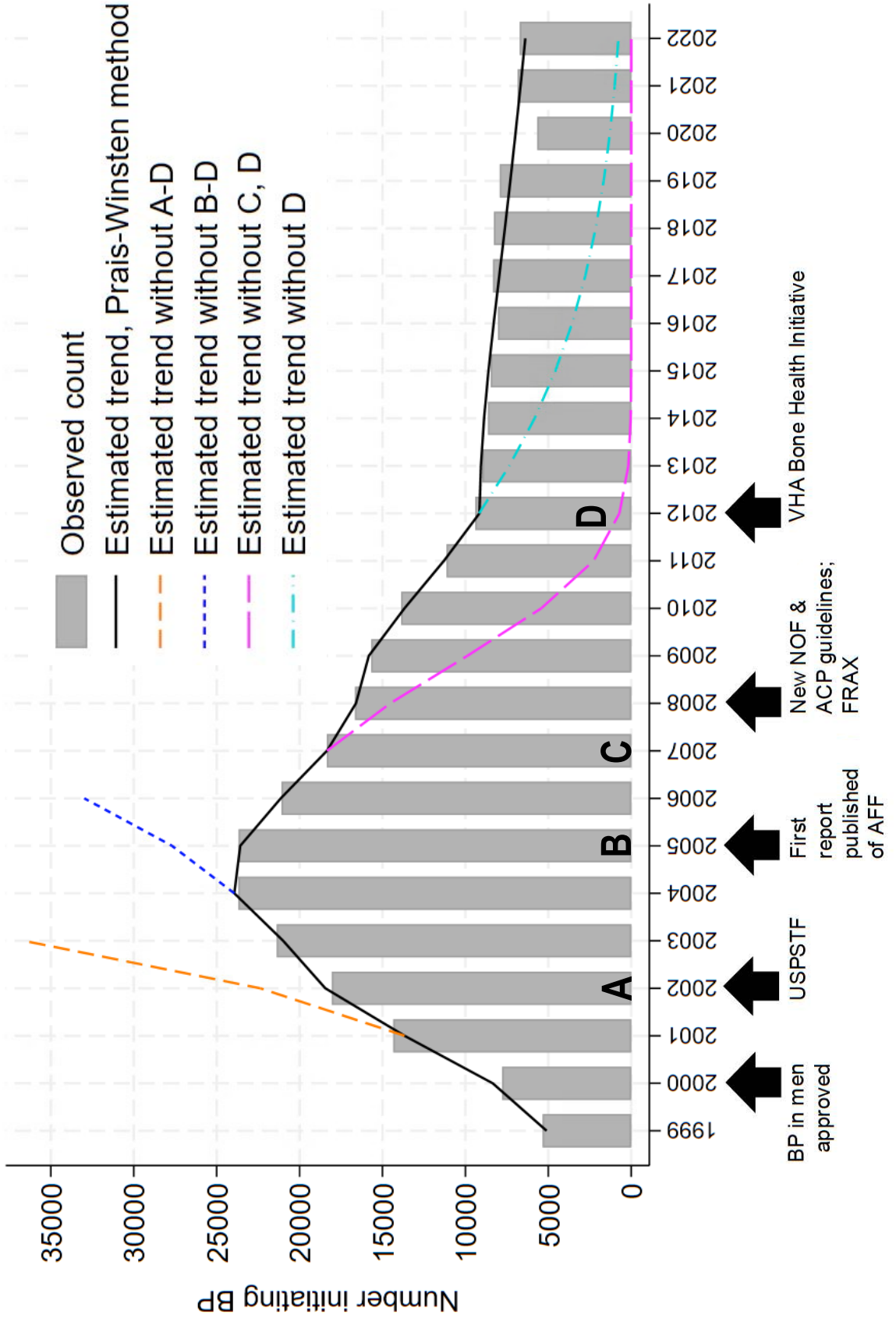

Supplement: Supplementary file 1 — (PDF 286 KB) [file 11657_2025_1635_MOESM1_ESM.pdf]

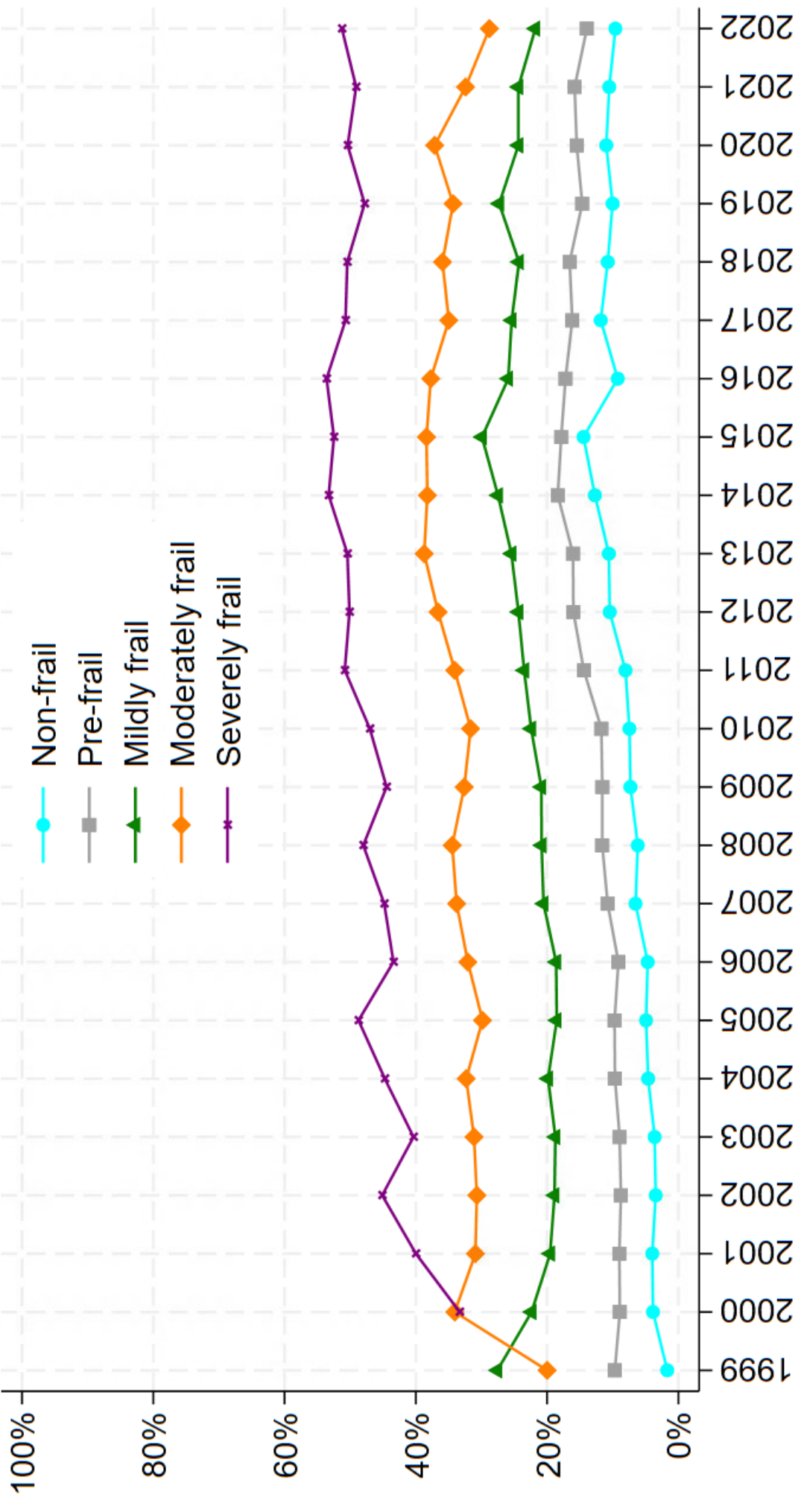

Supplement: Supplementary file 2 — (PDF 226 KB) [file 11657_2025_1635_MOESM2_ESM.pdf]
